# Perivascular inflammation in the progression of aortic aneurysms in Marfan syndrome

**DOI:** 10.1172/jci.insight.184329

**Published:** 2025-08-28

**Authors:** Hiroyuki Sowa, Hiroki Yagi, Kazutaka Ueda, Masaki Hashimoto, Kohei Karasaki, Qing Liu, Atsumasa Kurozumi, Yusuke Adachi, Tomonobu Yanase, Shun Okamura, Bowen Zhai, Norifumi Takeda, Masahiko Ando, Haruo Yamauchi, Nobuhiko Ito, Minoru Ono, Hiroshi Akazawa, Issei Komuro

**Affiliations:** 1 Department of Cardiovascular Medicine, Graduate School of Medicine,; 2 Marfan Syndrome Center, Graduate School of Medicine, and; 3 Department of Cardiac & Thoracic Surgery, Graduate School of Medicine, The University of Tokyo, Tokyo, Japan.; 4 Department of Cardiovascular Medicine, Kanto Central Hospital of the Mutual Aid Association of Public School Teachers, Tokyo, Japan.; 5 International University of Health and Welfare, Tokyo, Japan.; 6 Department of Frontier Cardiovascular Science, Graduate School of Medicine, The University of Tokyo, Tokyo, Japan.

**Keywords:** Cardiology, Inflammation, Cardiovascular disease

## Abstract

Inflammation plays important roles in the pathogenesis of vascular diseases. We here show the involvement of perivascular inflammation in aortic dilatation of Marfan syndrome (MFS). In the aorta of patients with MFS and *Fbn1*^C1041G/+^ mice, macrophages markedly accumulated in periaortic tissues with increased inflammatory cytokine expression. Metabolic inflammatory stress induced by a high-fat diet (HFD) enhanced vascular inflammation predominantly in periaortic tissues and accelerated aortic dilatation in *Fbn1*^C1041G/+^ mice, both of which were inhibited by low-dose pitavastatin. HFD feeding also intensifies structural disorganization of the tunica media in *Fbn1*^C1041G/+^ mice, including elastic fiber fragmentation, fibrosis, and proteoglycan accumulation, along with increased activation of TGF-β downstream targets. Pitavastatin treatment mitigated these alterations. For noninvasive assessment of perivascular adipose tissues (PVAT) inflammation in a clinical setting, we developed an automated analysis program for CT images using machine learning techniques to calculate the perivascular fat attenuation index of the ascending aorta (AA-FAI), correlating with periaortic fat inflammation. The AA-FAI was significantly higher in patients with MFS compared with patients without hereditary connective tissue disorders. These results suggest that perivascular inflammation contributes to aneurysm formation in MFS and might be a target for preventing and treating vascular events in MFS.

## Abstract 2

Perivascular inflammation contributes to aneurysm formation in Marfan syndrome and might be a novel target for preventing and treating vascular events in Marfan syndrome.

## Graphical abstract

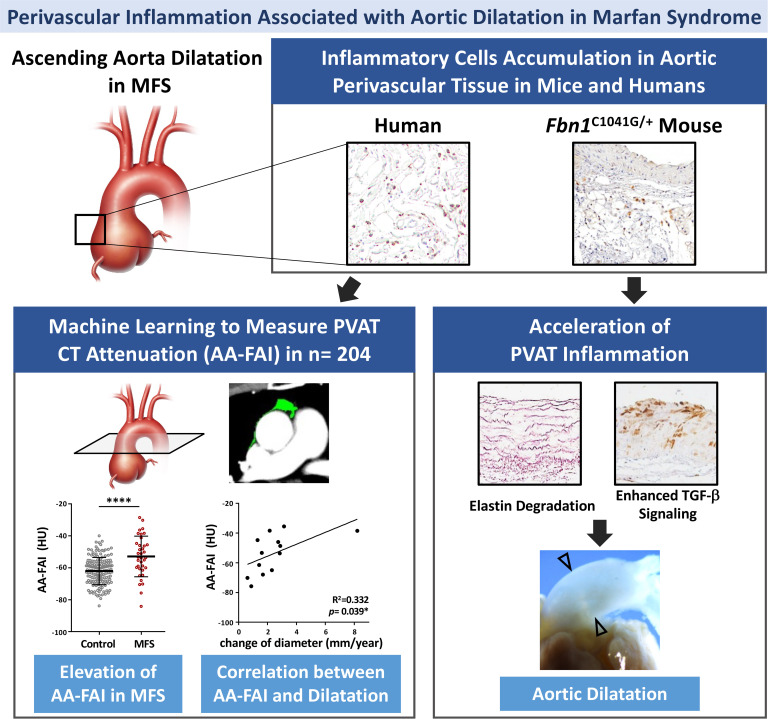

## Introduction

Marfan syndrome (MFS) is an autosomal-dominant inherited disorder caused by a genetic mutation in *FBN1* that results in systemic connective tissue abnormalities (1–4). Patients with MFS typically present with ocular, skeletal, and cardiovascular manifestations, with aortic root dilatation and subsequent aortic dissection or rupture being life-threatening complications (5–7). *FBN1* mutations reduce tissue integrity by interfering with the normal assembly of microfibrils and by activating TGF-β, a multifunctional cytokine involved in the proliferation, differentiation, and extracellular matrix (ECM) formation through the activation of extracellular signal-regulated kinases (ERKs), SMADs, and matrix metalloproteinases (MMPs), leading to increased elastin destruction and subsequent aortic dilatation (5, 6, 8, 9). Although drugs such as β-blockers and angiotensin receptor blockers (ARBs) slow the progression of aortic root dilatation (7, 10–14), it is difficult to stop the dilatation and long-term follow-up with serial imaging and aortic root replacement are crucial for the management of MFS (15–18). A better understanding of the pathogenesis of aortic disease in MFS and the development of novel therapeutic approaches have been awaited.

Inflammation plays a critical role in the development of vascular diseases (19–21). Recent studies have revealed the significant contribution of perivascular tissue inflammation to the development and progression of vascular diseases (22–26), and we have reported that inflammation of perivascular adipose tissues (PVAT) surrounding blood vessels mediates vascular remodeling after injury (27). In MFS, the presence of vascular inflammation in aortic disease has been demonstrated in patients and mice (28–31), with macrophage accumulation primarily in the adventitia (28, 32); however, inflammation in the PVAT and its contribution to structural abnormalities of the tunica media remain elusive. Since *FBN1* mutations have been reported to cause adipose tissue abnormalities, such as lipodystrophy or obesity (33), abnormal PVAT may be involved in the pathogenesis of aortic disease in MFS.

PVAT inflammation can be evaluated noninvasively through CT attenuation as measured by the perivascular fat attenuation index (FAI) (34). The FAI is a sensitive and dynamic biomarker of coronary inflammation as well as a strong and independent predictor of adverse cardiovascular events (34, 35); furthermore, the FAI in the abdominal aorta is reportedly associated with enlargement of abdominal aortic aneurysms (36). More recently, perivascular FAI around the ascending aorta (AA) has been reported to be higher in 3 consecutive patients with MFS than normal controls, while the volume of aortic PVAT tissue was lower in MFS (37), implying the feasibility of noninvasive assessment of the AA inflammation with FAI in MFS.

In this study, we evaluated the inflammation in tissues surrounding the aorta in humans and mice with MFS. We show pronounced inflammatory changes detected in the periaortic tissues in patients with MFS and *Fbn1*^C1041G/+^ mice and the involvement of the perivascular tissue inflammation in the progression of aortic dilatation. Furthermore, to measure the FAI of the AA (AA-FAI), we developed an automated image recognition program based on machine learning and analyzed PVAT inflammation caused by various types of aortic enlargement, including MFS.

## Results

### Predominant inflammatory response in periaortic tissues in MFS.

**Figure 1:**
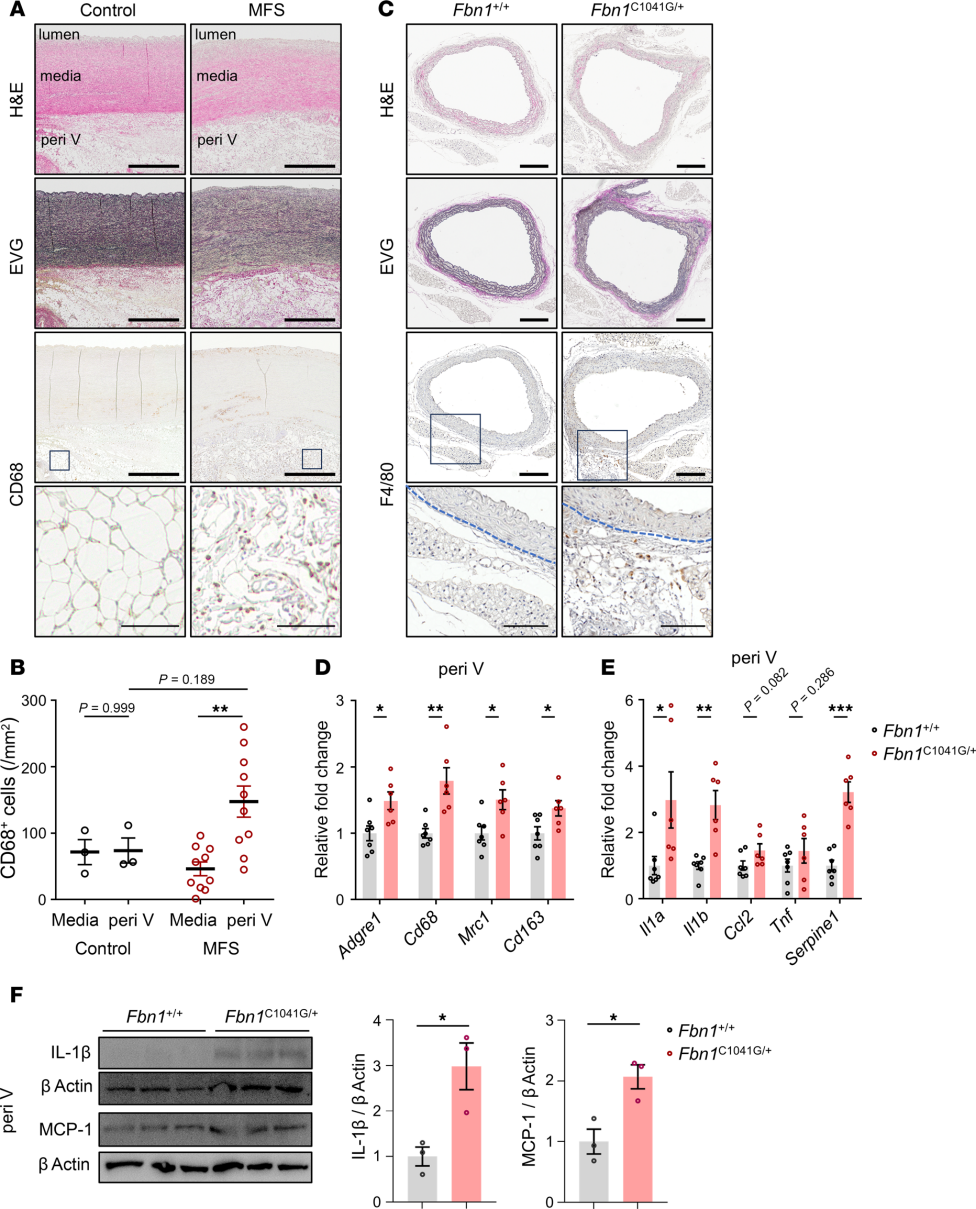
The periaortic inflammatory response of MFS mice and patients. (**A**) Histological analysis with H&E and Elastica van Gieson (EVG) staining and CD68 immunohistochemical staining in ascending aorta of patients with Marfan syndrome (MFS) and heart transplant recipients (control). Scale bars: 1,000 μm (thick bars) and 100 μm (thin bars). (**B**) The number of CD68^+^ cells/mm^2^ in media and perivascular (peri V) area was calculated in each group (control, *n* = 3; MFS, *n* = 10). The data are presented as mean ± SD. **P < 0.01, 1-way ANOVA with Tukey’s multiple-comparison test. (**C**) Histological analysis with H&E and EVG staining and F4/80 immunohistochemical staining in ascending aorta of *Fbn1*^+/+^ and *Fbn1*^C1041G/+^ mice (24 weeks of age). Scale bars: 200 μm (thick bars) and 100 μm (thin bars). Blue dashed lines indicate the external elastic lamina. (**D**) The mRNA expressions of marker genes of macrophages in the peri V tissues of *Fbn1*^+/+^ and *Fbn1*^C1041G/+^ mice (24 weeks of age) (*n* = 6–7). The data are presented as fold induction over control. The results were systematically normalized using glyceraldehyde-3-phosphate dehydrogenase (*Gapdh*). The data are presented as mean ± SEM. *P < 0.05, **P < 0.01, unpaired 2-tailed *t* test with Welch’s correction. (**E**) The mRNA expressions of marker genes of inflammatory cytokines in the peri V tissues of *Fbn1*^+/+^ and *Fbn1*^C1041G/+^ mice (24 weeks of age) (*n* = 6–7). The data are presented as fold induction over control. The results were systematically normalized using *Gapdh*. The data are presented as mean ± SEM. *P < 0.05, **P < 0.01, ***P < 0.001, unpaired 2-tailed *t* test with Welch’s correction. (**F**) Immunoblot analysis of IL-1β, MCP-1, and β actin in the peri V tissues of *Fbn1*^+/+^ and *Fbn1*^C1041G/+^ mice (24 weeks of age). The quantifications of IL-1β/β actin (*n* = 3) and MCP-1/β actin (*n* = 3) are shown as bar graphs. The data are presented as mean ± SEM. *P < 0.05, unpaired 2-tailed *t* test with Welch’s correction.

To determine the primary location of inflammation in MFS aortic disease, we first evaluated the accumulation of immune cells in aortic root specimens from patients with MFS. IHC staining for CD68 revealed marked accumulation of macrophages in perivascular tissues, especially in PVAT, at the aortic root (Figure 1A). Accumulation of CD68^+^ cells was also observed in the adventitia and the intima; however, their accumulation in the tunica media was less than that in perivascular tissues (Figure 1, A and B). CD68^+^ cell accumulation in periaortic tissues of patients with MFS tended to be greater than that in those of control patients without MFS (Figure 1, A and B). Consistently, in mice with *Fbn1* missense mutation p.(Cys1041Gly), more F4/80^+^ macrophages accumulated in periaortic tissues compared with that in *Fbn1^+/+^* control WT mice (Figure 1C). In the perivascular tissues of *Fbn1*^C1041G/+^ mice, the expression levels of marker genes of macrophages and inflammatory cytokines such as *Il1a*, *Il1b*, and *Serpine1* (Pai1) were significantly upregulated (Figure 1, D and E). In the perivascular tissues of *Fbn1*^C1041G/+^ mice, the protein expression of IL-1β and MCP-1 was also significantly upregulated compared with *Fbn1^+/+^* mice (Figure 1F). These results suggest that, in MFS aortopathy, there was an enhanced inflammatory response in the perivascular tissue.

### Inflammation in PVAT causes aortic dilatation in MFS.

**Figure 2:**
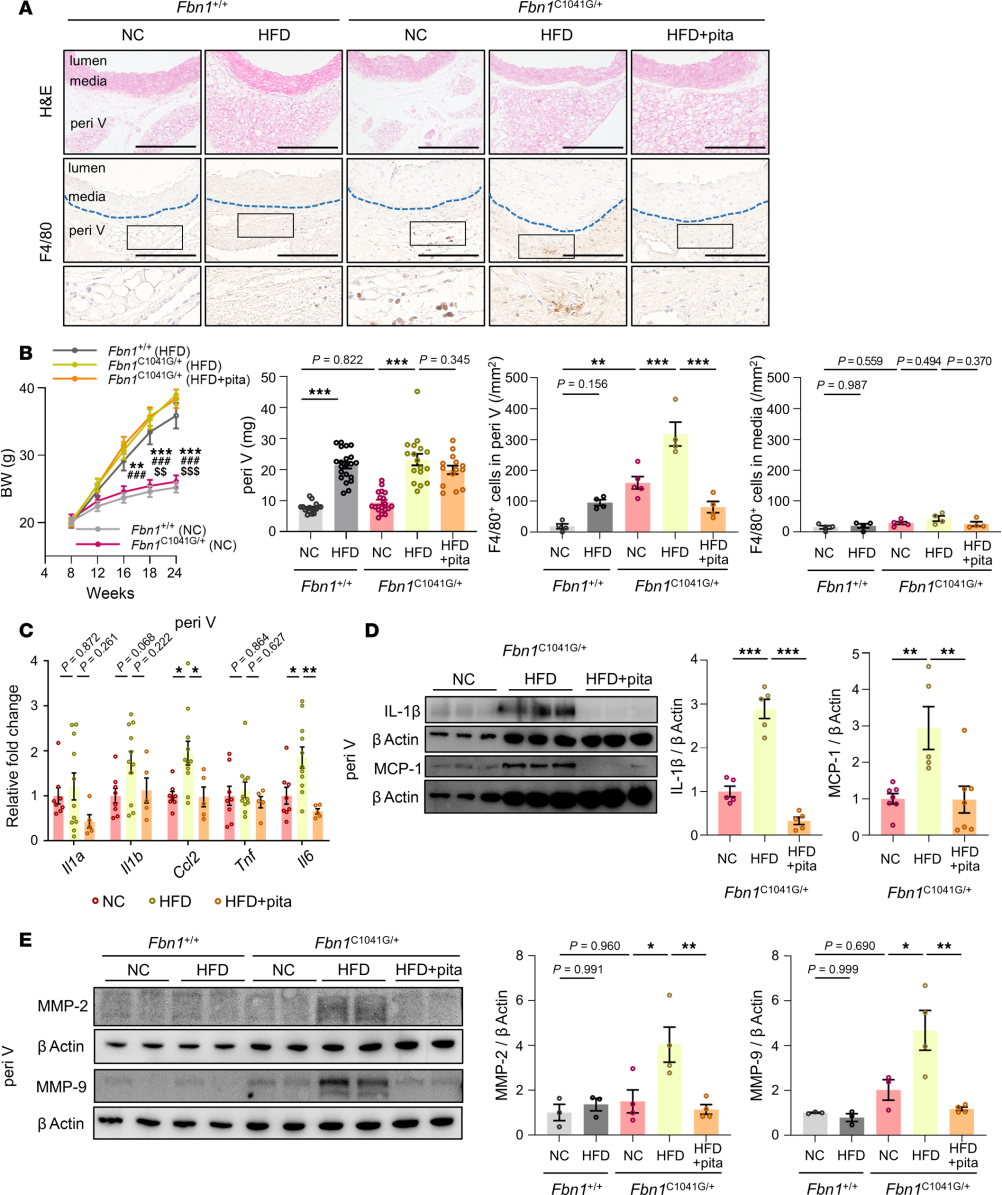
Inflammation in PVAT induced by metabolic stress accelerated aortic dilatation in *Fbn1*^C1041G/+^ mice. (**A**) Hematoxylin–eosin (H&E) and immunohistochemical staining for F4/80 in ascending aorta of *Fbn1*^+/+^ and *Fbn1*^C1041G/+^ mice (24 weeks of age) receiving normal chow (NC), high fat diet (HFD), or HFD with pitavastatin (pita). Scale bars: 200 μm. Blue dashed lines indicate the external elastic lamina. (**B**) Time-coursed changes of body weight in *Fbn1*^+/+^ (NC, *n* = 20; HFD, *n* = 20) and *Fbn1*^C1041G/+^ (NC, *n* = 19; HFD, *n* = 24; HFD with pita, *n* = 24) mice. **P < 0.01, ***P < 0.001 (NC in *Fbn1*^C1041G/+^ vs HFD in *Fbn1*^C1041G/+^), ^###^*P* < 0.001 (NC in *Fbn1*^C1041G/+^ vs HFD + pita in *Fbn1*^C1041G/+^), ^$$^*P* < 0.01, ^$$$^*P*<0.001 (NC in *Fbn1*^C1041G/+^ vs HFD in *Fbn1*^+/+^), 2-way ANOVA followed by Tukey’s multiple-comparisons. . Weight of the perivascular tissues (peri V) (*n* = 14 to 21) and the number of F4/80-positive cells in the peri V and media (n = 4 to 5) of *Fbn1*^+/+^ and *Fbn1*^C1041G/+^ mice (24 weeks of age) receiving NC, HFD, or HFD with pita was calculated. **P < 0.01, ***P < 0.001, 1-way ANOVA with Tukey's multiple comparisons. (**C**) The mRNA expressions of inflammatory cytokines in the peri V (*n* = 5 to 11). The results were normalized using β-2 microglobulin (*B2m*). *P < 0.05, **P < 0.01, 1-way ANOVA with Tukey’s multiple-comparisons. (**D**) Immunoblot analysis of IL-1β, MCP-1, and β actin in the peri V. The quantifications of IL-1β/β actin (*n* = 5) and MCP-1/β actin (*n* = 5 to 7) are shown. **P < 0.01, ***P < 0.001, 1-way ANOVA with Tukey’s multiple-comparisons. (**E**) Immunoblot analysis of MMP-2, MMP-9, and β actin in the peri V. The quantifications of MMP-2/β actin (*n* = 3 to 4) and MMP-9/β actin (*n* = 3 to 4) are shown as bar graphs. *P < 0.05, **P < 0.01, 1-way ANOVA with Tukey’s multiple-comparison test. All data are presented as mean ± SEM.

**Figure 3:**
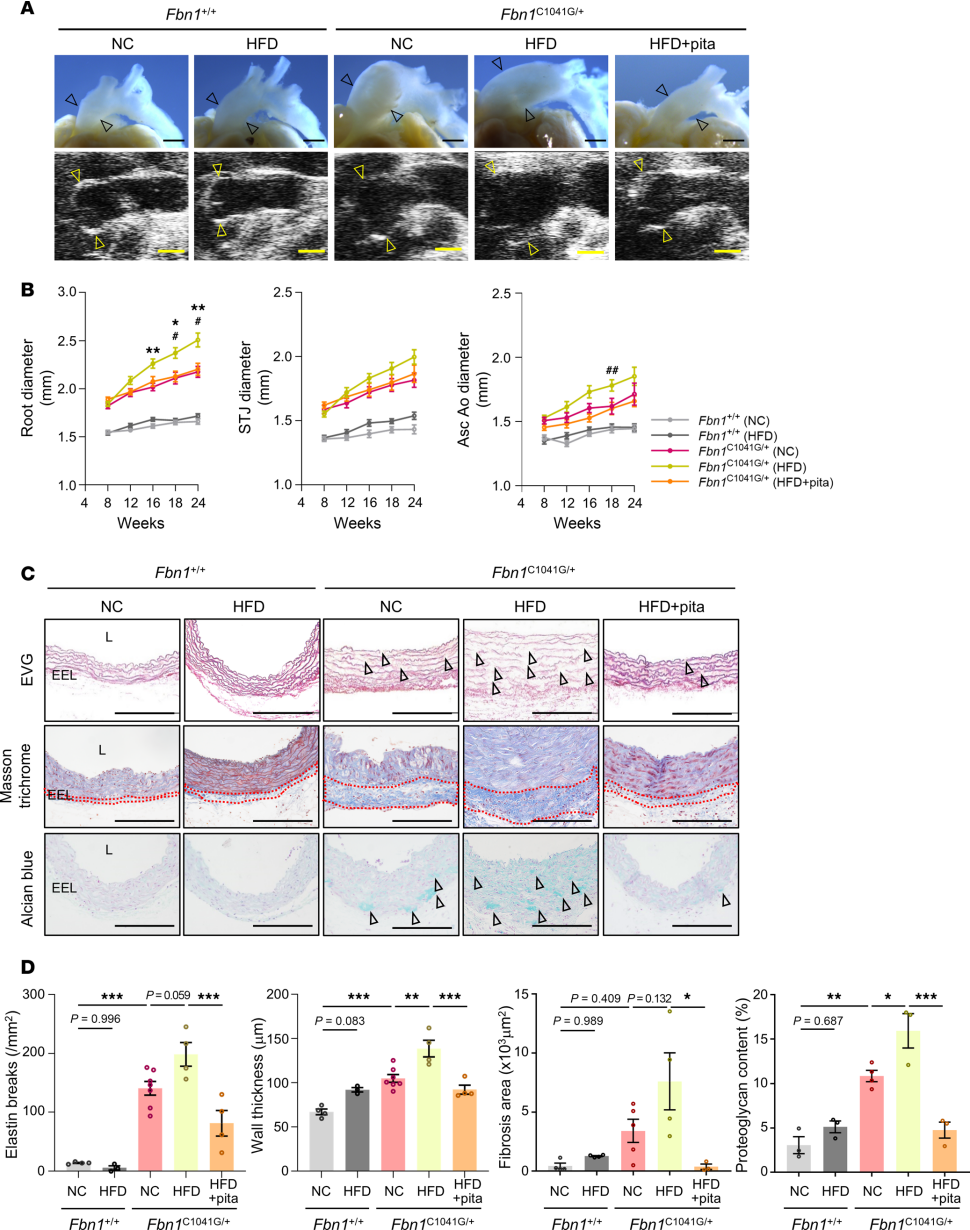
Pathological changes in the aorta of *Fbn1*^C1041G/+^ mice induced by PVAT inflammation. (**A**) Macroscopic appearance and ultrasound images of aorta in *Fbn1*^+/+^ and *Fbn1*^C1041G/+^ mice (24 weeks of age) receiving normal chow (NC), high-fat diet (HFD), or HFD with pitavastatin (pita). Arrowheads indicate ascending aorta (upper panel; black) and aortic root (lower panel; yellow). Scale bars: 1,000 μm. (**B**) Time-coursed changes (8–24 weeks of age) of aortic root, sinotublar junction (STJ) and ascending aorta diameter in *Fbn1*^+/+^ (NC, *n* = 20; HFD, *n* = 20) and *Fbn1*^C1041G/+^ (NC, *n* = 19; HFD, *n* = 24; HFD with pita, *n* = 24) mice. The data are presented as mean ± SEM. *P < 0.05 (NC vs HFD in *Fbn1*^C1041G/+^), **P < 0.01 (NC vs HFD in *Fbn1*^C1041G/+^), ^#^*P* < 0.05 (HFD vs HFD with pita in *Fbn1*^C1041G/+^), ^##^*P* < 0.01 (HFD vs HFD with pita in *Fbn1*^C1041G/+^), 2-way ANOVA followed by Tukey’s multiple-comparison test. (**C**) Histological analysis with elastic van Gieson (EVG) staining, Masson’s trichrome staining, and Alcian blue staining in ascending aorta of *Fbn1*^+/+^ and *Fbn1*^C1041G/+^ mice (24 weeks of age) receiving NC, HFD, or HFD with pita. Scale bars: 200 μm. L; lumen, EEL; external elastic lamina. (**D**) Measurement of aortic wall thickness, aortic wall architecture indicated by the number of breaks of the elastic fiber per mm^2^, fibrosis area, and proteoglycan content in ascending aorta of *Fbn1*^+/+^ and *Fbn1*^C1041G/+^ mice (24 weeks of age) receiving NC, HFD, or HFD with pita (*n* = 3–7). The data are presented as mean ± SEM. *P < 0.05, **P < 0.01, ***P < 0.001, 1-way ANOVA with Tukey’s multiple-comparison test.

To assess the involvement of periaortic inflammation in the development of aortic dilatation in MFS, we induced PVAT inflammation in *Fbn1*^C1041G/+^ mice by applying metabolic stress via high-fat diet (HFD) feeding (38, 39). A 16-week feeding regimen consisting of HFD induced the accumulation of F4/80^+^ cells and upregulation of mRNA and protein expression levels of various inflammatory markers and MMPs in perivascular tissues of *Fbn1*^C1041G/+^ mice (Figure 2, A–E). Along with increased PVAT inflammation, the diameter of the aortic root significantly increased in *Fbn1*^C1041G/+^ mice on an HFD regimen compared with those fed with a normal chow (NC) diet (Figure 3, A and B). Pitavastatin (0.3 mg/kg/day), which exerts antiinflammatory effects on adipose tissues (40), suppressed HFD-induced accumulation of F4/80^+^ cells, mRNA and protein expression of inflammatory cytokines and MMPs in perivascular tissues, and the expansion of the aortic root (Figure 2, A–E, and Figure 3, A and B). The expansion of the ascending aorta was partially suppressed in pitavastatin-treated HFD-fed *Fbn1*^C1041G/+^ mice, as compared with HFD-fed *Fbn1*^C1041G/+^ mice (Figure 3, A and B). The dose of pitavastatin used in this study did not affect the metabolic profiles—including serum cholesterol, blood sugar levels, or blood pressure—of HFD-fed *Fbn1*^C1041G/+^ mice (Supplemental Figure 1; supplemental material available online with this article; https://doi.org/10.1172/jci.insight.184329DS1). Additionally, pitavastatin exhibited a trend toward suppression of inflammation in the perivascular tissues of NC-fed *Fbn1*^C1041G/+^ mice, as evidenced by reduced accumulation of F4/80^+^ cells and decreased mRNA and protein expression levels of inflammatory cytokines, although these differences did not reach statistical significance (Supplemental Figure 2). These results suggest a strong correlation between PVAT inflammation and aortic dilatation in MFS, independent of changes in metabolic profiles.

**Figure 4:**
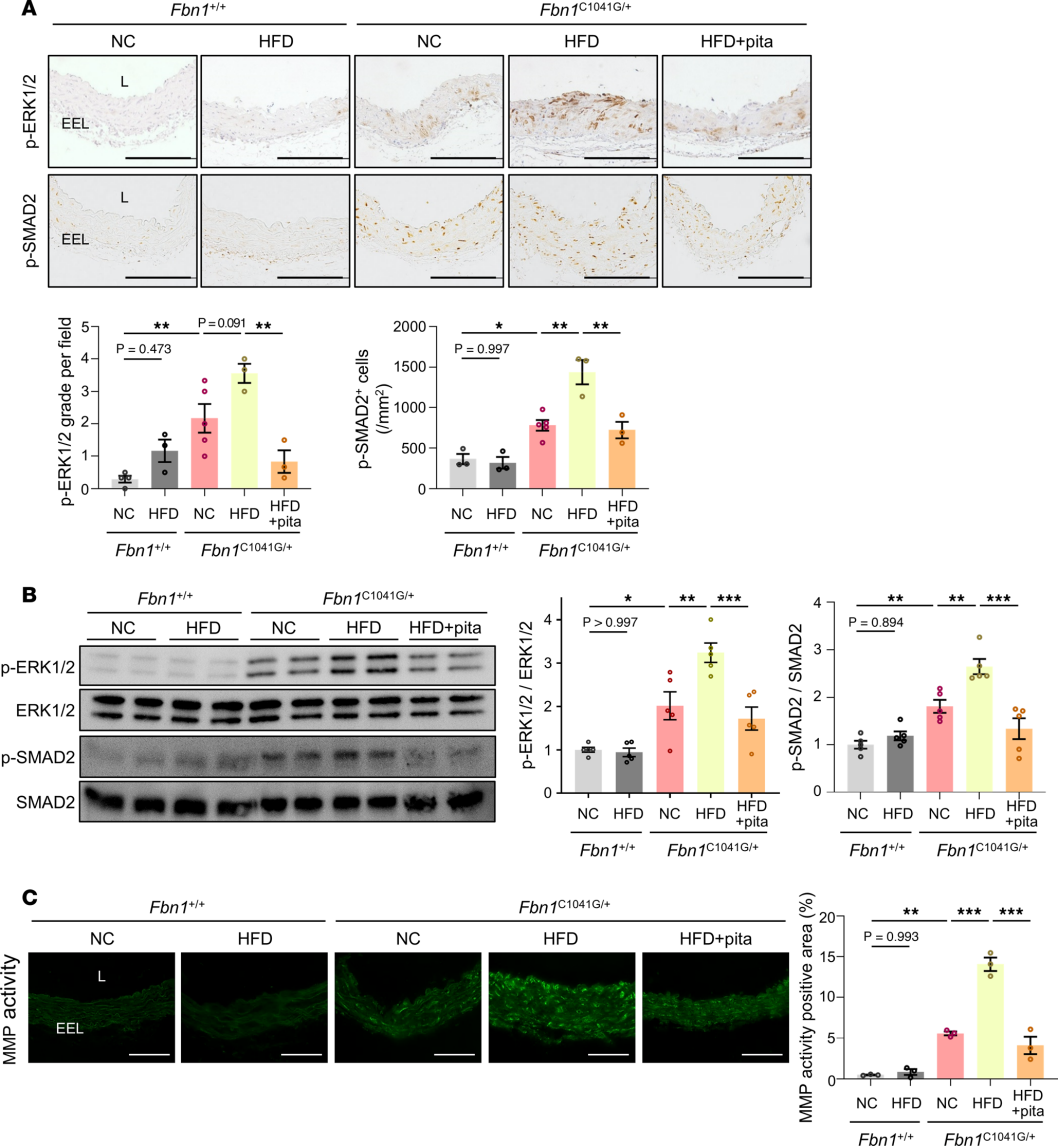
TGF-β downstream changes in the aorta of *Fbn1*^C1041G/+^ mice induced by PVAT inflammation. (**A**) Immunostaining for phosphorylated-ERK1/2 (p-ERK1/2), and phosphorylated-SMAD2 (p-SMAD2), in ascending aorta of *Fbn1*^+/+^ and *Fbn1*^C1041G/+^ mice (24 weeks of age) receiving normal chow (NC), high-fat diet (HFD), or HFD with pitavastatin (pita). Scale bars: 200 μm. L; lumen, EEL; external elastic lamina. The quantifications of p-ERK1/2 (*n* = 3 to 5) and p-SMAD2 (*n* = 3 to 5) are shown as bar graphs. The data are presented as mean ± SEM. *P < 0.05, **P < 0.01, 1-way ANOVA with Tukey’s multiple-comparison test. (**B**) Immunoblot analysis of p-ERK1/2, ERK1/2, p-SMAD2, SMAD2 in ascending aorta of *Fbn1*^+/+^ and *Fbn1*^C1041G/+^ mice (24 weeks of age) receiving NC, HFD, or HFD with pita. The quantifications of p-ERK1/2/ERK1/2 (*n* = 5) and p-SMAD2/SMAD2 (*n* = 5) are shown as bar graphs. The data are presented as mean ± SEM. *P < 0.05, **P < 0.01, ***P < 0.001, 1-way ANOVA with Tukey’s multiple-comparison test. (**C**) In situ zymography for gelatinase activity in ascending aorta of *Fbn1*^+/+^ and *Fbn1*^C1041G/+^ mice (24 weeks of age) receiving NC, HFD, or HFD with pita. Scale bars: 100 μm. The quantification of MMP activity (*n* = 3) is shown as bar graphs. The data are presented as mean ± SEM. **P < 0.01, ***P < 0.001, 1-way ANOVA with Tukey’s multiple-comparison test.

We subsequently analyzed the pathological changes in the vascular structure of HFD-fed MFS mice. *Fbn1*^C1041G/+^ mice showed elastic fiber fragmentation and disorganization, fibrosis, and proteoglycan accumulation compared with *Fbn1^+/+^* mice as shown by Elastica-Van Gieson (EVG), Masson trichrome, and Alcian blue staining, respectively (Figure 3, C and D). Notably, these features were more pronounced in HFD-fed *Fbn1*^C1041G/+^ mice than in NC-fed mice (Figure 3, C and D). Similarly, expression of phosphorylated ERK1/2 and SMAD2, and MMP activity in the vascular medial layer, increased in *Fbn1*^C1041G/+^ mice compared with *Fbn1^+/+^* mice and was enhanced by HFD feeding, and these changes were significantly attenuated by pitavastatin treatment (Figure 4, A–C). These results suggest that HFD feeding exacerbates structural disorganization of the tunica media with the enhanced activation of TGF-β downstream targets in MFS mice.

### Evaluation of aortic PVAT inflammation in patients with MFS via a noninvasive approach.

**Figure 5:**
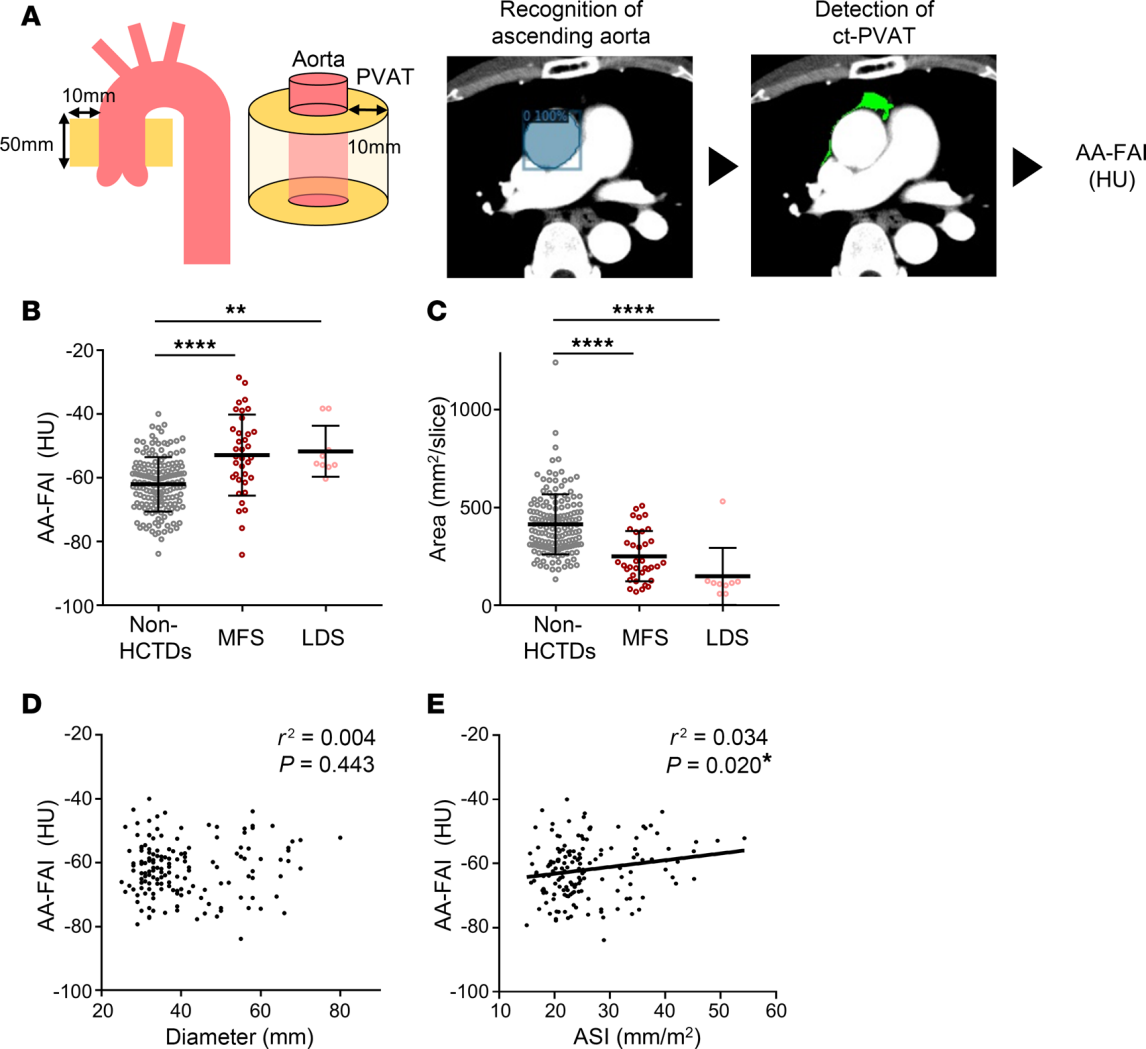
Noninvasive evaluation of aortic PVAT inflammation in MFS. (**A**) Procedure for calculating the fat attenuation index to the ascending aorta (AA-FAI). Machine learning–based program recognizes the ascending aorta, selects areas with CT attenuation corresponding to fat tissue within a 10 mm range around the vessel wall, defines them as perivascular adipose tissues (PVAT) on CT (ct-PVAT), and outputs the average values of attenuation of all pixels in the ct-PVAT as the AA-FAI. (**B** and **C**) The AA-FAI (**B**) and the area of ct-PVAT (**C**) was calculated in each group (non-HCTDs, *n* = 159; MFS, *n* = 36; LDS, *n* = 9). The data are presented as mean ± SD. **P < 0.01, ****P < 0.0001, 1-way ANOVA with Tukey’s multiple-comparison test. (**D**) Scatterplots showing the correlation between the aortic diameter and AA-FAI in patients without HCTDs (*n* = 159). (**E**) Scatterplots showing the correlation between the aortic size index (ASI) and AA-FAI in patients without HCTDs (*n* = 159). HCTDs, hereditary connective tissue disorders; HFD, high-fat diet; LDS, Loeys-Dietz Syndrome; MFS, Marfan syndrome.

To understand the relationship between aortic enlargement and PVAT inflammation in patients with MFS, we adopted FAI, a potentially novel marker for coronary vascular inflammation (34, 35, 41), for the noninvasive evaluation of PVAT inflammation of the aorta. Since, in a small case series, perivascular FAI around the ascending aorta was elevated in patients with MFS compared with controls, and the amount of aortic PVAT tissue was decreased (37), we employed machine learning techniques to develop an automated analysis program for calculating the FAI of the ascending aorta (AA-FAI) on a larger scale. The program recognizes the AA, selects areas with CT attenuation corresponding to fat tissue within a 10 mm range around the vessel wall, defines them as PVAT on CT (ct-PVAT), and outputs the average values of attenuation of all pixels in the ct-PVAT as the AA-FAI (Figure 5A). The program correctly identified the AA in 94.4% of cases and successfully calculated the AA-FAI. We collected and analyzed CT imaging data and background information from 204 patients; 45 with hereditary connective tissue disorders (HCTDs), consisting of MFS (*n* = 36) and Loeys–Dietz Syndrome (LDS) (*n* = 9), and 159 without HCTDs (Supplemental Figure 3). All CT images of patients with HCTDs were from those scheduled for aortic surgery, and patients without HCTDs had none, mild, or severe dilatation with surgical indications (*n* = 69, 55, 35, respectively). The characteristics of patients in each group are shown in Table 1 and Supplemental Table 2.

AA-FAI was significantly higher in patients with MFS than in those without HCTDs (non-HCTDs, –62.02 ± 8.54 Hounsfield unit [HU] vs MFS, –52.90 ± 12.69 HU; *P* < 0.0001) (Figure 5B), while the area of the ct-PVAT was smaller in patients with MFS than in those without HCTDs (non-HCTDs, 414.10 ± 153.31 mm^2^/slice, vs MFS, 251.19 ± 127.89 mm^2^/slice; *P* < 0.0001) (Figure 5C). These differences were also observed between the LDS and non-HCTDs patient groups (non-HCTDs, –62.02 ± 8.54 HU, vs LDS, –51.70 ± 7.99 HU, *P* < 0.0001; non-HCTDs, 414.10 ± 153.31 mm^2^/slice, vs LDS, 148.35 ± 145.35 mm^2^/slice, *P* < 0.0001) (Figure 5, B and C). While no significant association was observed between aortic diameter and AA-FAI in patients without HCTDs (Figure 5D), a significant correlation was found between the aortic size index (ASI), which adjusts aortic diameter for body surface area, and AA-FAI (Figure 5E), suggesting a link between aortic enlargement and increased AA-FAI. Notably, the AA-FAI in patients with MFS was significantly higher than in patients without HCTDs, even when compared with those with advanced aortic enlargement (≥50 mm) or those in the highest ASI tertile (Supplemental Figure 4, A and B). These findings indicate that aortic PVAT inflammation increases with aortic enlargement in patients without HCTDs, whereas it is more pronounced in those with HCTDs.

### Clinical relevance of PVAT inflammation assessment in MFS.

**Figure 6:**
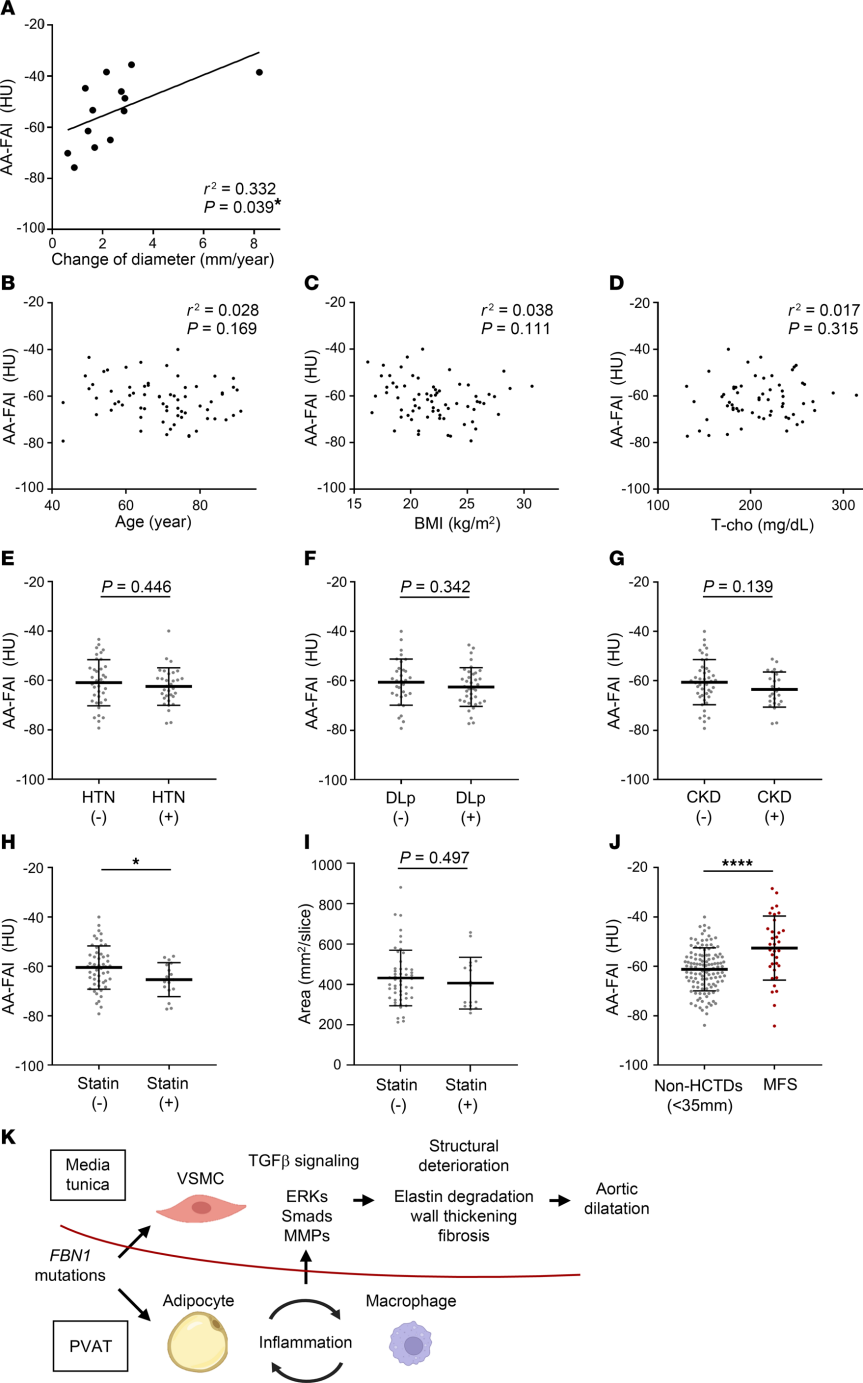
Effect of background factors on AA-FAI. (**A**) Scatterplots showing the correlation between the expansion speed of the aortic diameter and the AA-FAI in the 2 years leading to prophylactic aortic surgery (*n* = 13). Patients with at least 2 follow-ups for echo cardiography within the last 2 years of imaging the analyzed CT were recruited. (**B**–**D**) Scatterplots showing the correlation between age, BMI, total cholesterol (T-chol), and the fat attenuation index to the ascending aorta (AA-FAI) in patients without enlargement of ascending aorta (age and BMI, *n* = 69; T-chol, *n* = 61). (**E**–**G**) Among the patients without enlargement of ascending aorta, the AA-FAI was compared in 2 groups according to the absence or presence of hypertension (HTN) [(–), *n* = 37; (+), *n* = 32], dyslipidemia (DLp) [(–), *n* = 32; (+), *n* = 37], and chronic kidney disease (CKD) [(–), *n* = 44; (+), *n* = 25]. The data are presented as mean ± SD. Unpaired 2-tailed *t* test with Welch’s correction. (**H** and **I**) Among the patients without enlargement of ascending aorta, the AA-FAI and ct-PVAT area were compared in 2 groups according to the absence or presence of statin treatment [(–), *n* = 52; (+), *n* = 17]. The data are presented as mean ± SD. *P < 0.05, unpaired 2-tailed *t* test with Welch’s correction. (**J**) The AA-FAI was compared in patients with non-HCTDs and MFS who were not taking statins (non-HCTDs, *n* = 121; MFS, *n* = 34). The data are presented as mean ± SD. ****P < 0.0001, unpaired 2-tailed *t* test with Welch’s correction. (**K**) The scheme of MFS pathology suggested by this study. Perivascular adipose tissue (PVAT) inflammation enhances TGF-β signaling in the tunica media and leads to aortic dilatation. Immune cells including macrophages that migrate into perivascular tissues due to signals of inflammatory adipokines may secret TGF-β that further enhances signaling in the tunica media. ERKs, extracellular signal-regulated kinases; HCTDs, hereditary connective tissue disorders; HFD, high-fat diet; LDS, Loeys-Dietz Syndrome; MFS, Marfan syndrome; MMPs, matrix metalloproteinases; VSMC, vascular smooth muscle cell.

There was a positive correlation between the expansion speed of the aortic diameter measured by ultrasound imaging and the AA-FAI in the 2 years before aortic surgery (Figure 6A). The receiver operating characteristic (ROC) analysis of the AA-FAI prediction for HCTDs or MFS is shown in Supplemental Figure 5. The AUC was 0.74 (95% CI, 0.66–0.83) and 0.73 (95% CI, 0.63–0.83) for HCTDs and MFS, respectively. AA-FAI had a highly sensitive and specific predictive value for MFS, with an optimal cutoff of –55.3 HU (sensitivity, 0.61; specificity, 0.81). These results suggest that aortic PVAT inflammatory status is correlated with the rate of aortic enlargement and that AA-FAI may be a potential specific biomarker for HCTDs.

As characteristics differ significantly between patients with HCTDs and without HCTDs (Table 1), we then examined the effect of these characteristics on the AA-FAI. For factors such as, age, BMI, hypertension, dyslipidemia, statin use, total cholesterol level, and chronic kidney disease (CKD), propensity score matching did not provide sufficient sample size to perform adequate statistical analysis. Hence, data from the patients without HCTDs who have normal aortic diameter (<35 mm) were used to analyze the effect of each factor on the AA-FAI. No statistical association was found between age, BMI, or total cholesterol level and the AA-FAI (Figure 6, B–D). Furthermore, the AA-FAI was not correlated with hypertension, dyslipidemia, or CKD (Figure 6, E–G). Interestingly, the AA-FAI was lower in the statin use group (non-statin use, –60.46 ± 8.76 HU, vs statin use, –65.37 ± 6.84 HU; *P* = 0.039) (Figure 6H), while statin did not affect ct-PVAT area (non-statin use, 431.7 ± 138.2 mm^2^/slice, vs statin use, 406.3 ± 128.4 mm^2^/slice; *P* = 0.493) (Figure 6I). Patients with MFS still demonstrated higher AA-FAI than patients without HCTDs, even after excluding statin users from the analysis (non-HCTDs, –61.22 ± 8.69 HU, vs MFS, –52.61 ± 13.00 HU; *P* < 0.0001) (Figure 6J).

## Discussion

Inflammation in perivascular tissue is involved in the pathophysiology of various vascular diseases, including atherosclerosis and hypertension (22–25, 27). This study reveals a profound link between PVAT inflammation and the aortic disease of MFS. In both patients with MFS and *Fbn1*^C1041G/+^ mice, marked accumulation of macrophages and increased inflammatory cytokine expression were observed in periaortic tissues. In *Fbn1*^C1041G/+^ mice, HFD feeding enhanced perivascular inflammation and aortic dilatation, both of which were inhibited by low-dose pitavastatin without affecting lipid parameters in the blood. In humans, noninvasive evaluation using CT revealed that PVAT inflammation is more prominent in patients with HCTDs, including MFS and LDS, than in those without HCTDs. Furthermore, a higher degree of PVAT inflammation correlated with faster aortic enlargement in patients with MFS. Our findings suggest a close relationship between PVAT inflammation and aortic dilatation in MFS.

Many studies have demonstrated that *FBN1* mutations in MFS activate TGF-β signaling across tissues (42). In aortic smooth muscle cells of patients with MFS, activation of TGF-β signaling induces phosphorylation and activation of downstream targets, such as ERKs and SMADs, and increases expression of MMPs, which degrade elastin, leading to aortic dilatation and dissection (5, 6, 8, 9). This study demonstrated that enhancement of the inflammatory response via HFD-induced metabolic stress on PVAT in *Fbn1*^C1041G/+^ mice further activated TGF-β downstream targets in the tunica media and aggravated aortic dilatation. This implies that PVAT inflammation enhances TGF-β signaling in the tunica media and leads to aortic dilatation. Immune cells such as macrophages that migrate to perivascular tissues in response to PVAT-derived inflammatory adipokines such as MCP1 may produce TGF-β, further enhancing its signaling in the adjacent tunica media (Figure 6K).

FAI, which was analyzed using CT images, was recently developed to noninvasively assess PVAT inflammation. In patients with acute coronary syndrome, FAI was increased in PVAT around coronary arteries with plaque rupture compared with those in unruptured sites, which also correlated with ^18^F-fluorodeoxyglucose–positron emission tomography uptake (34). This method has been utilized in various vessels, including the coronary and carotid arteries and abdominal aorta, for the evaluation of inflammation in PVAT (34, 36, 43). In this study, we evaluated FAI in aortic PVAT and found that PVAT inflammation is more pronounced in patients with MFS than in those without HCTDs. A recent study reported that the ASI, which adjusts aortic diameter for body surface area, correlates with aortic FAI (44). Consistently, our data from patients without HCTDs also demonstrate a similar association. Interestingly, however, patients with HCTDs exhibited even higher AA-FAI than patients without HCTDs with large aortic diameters or high ASI. These findings suggest that a distinct mechanism may underly PVAT inflammation in MFS and that AA-FAI could serve as a diagnostic biomarker for HCTDs. Given that TGF-β signaling induces adipose dysfunction (33), the enhanced TGF-β signaling in MFS may directly mediate PVAT inflammation, and alternatively, as mechanical stress reportedly promotes adipocyte inflammation (33, 45, 46), MFS-induced elastin degradation and structural abnormalities in the aortic ECM may induce PVAT inflammation through enhanced mechanical stress on aorta. Further studies are needed to elucidate how *FBN1* mutations lead to adipose tissue inflammation.

While this study reveals a close association between PVAT inflammation and aortic dilatation in MFS, further investigation is warranted to establish the causal relationship. The mouse models employed in this study may lack sufficient specificity to validate the importance of PVAT inflammation. Neither HFD nor pitavastatin can be ruled out as drugs that target PVAT inflammation exclusively, as they both affect a wide variety of organs, including direct effects on plaque formation and TGF-β signaling within the vasculature (47, 48). Of note, it has been reported that PVAT is selectively depleted in SM22α^CreKI^/PPAR-γ^fl/fl^ mice (49), indicating that experimental approaches utilizing these mice may provide valuable insights into this matter. Our observation that statins suppress aortic dilatation in MFS mice aligns with a previous report (50), and there are reports indicating the pleiotropic effect of statins in human aortic aneurysms (47, 51). Verification of the effectiveness of statins in aortic dilatation in human MFS is awaited.

In conclusion, in MFS, marked inflammation occurs in perivascular tissues, which is involved in the progression of aortic dilatation. Currently, although β-blockers and ARBs are expected to slow the progression of aortic dilatation, they do not completely inhibit disease progression. Agents targeting perivascular tissue inflammation, such as statins, may be a promising treatment option to attenuate aortic dilatation in MFS. Research focusing on perivascular tissue inflammation will provide us with a better understanding of the pathogenesis of aortic disease in MFS that may lead to the development of innovative diagnostic and therapeutic strategies.

## Methods

### Sex as a biological variable.

Only male *Fbn1*^C1041G/+^ mice were used in this study because they develop more pronounced aortic phenotypes than females, facilitating clearer assessment of disease progression and treatment effects. This approach is consistent with prior studies using this model. However, whether the findings are applicable to female mice requires further investigation. In the human analyses, both sexes were included, but sex was not analyzed as a biological variable due to the clinical referral–based study design.

### Animal experiments.

Mice were housed in groups of 3–4 animals per cage with a 12-hour light–dark cycle at constant room temperature of 22°C ± 1°C with a humidity of 50% ± 15%. All were fed a standard chow (CE-2; Clea Japan Inc.) and water ad libitum during the experimental period. *Fbn1*^C1041G/+^ mice were obtained from the Jackson Laboratory (52) and maintained on a C57BL/6J background. *Fbn1*^+/+^ and *Fbn1*^C1041G/+^ mice fed with a High Fat Diet32 (32% fat, HFD32) and a matched control diet (4.8% fat, CE-2), both purchased from Clea Japan Inc. The diets were provided during the age of 8–24 weeks for the *Fbn1*^+/+^ and *Fbn1*^C1041G/+^ mice and the experimental endpoints were 24 weeks, respectively. Pitavastatin (NK-104, Kowa Co. Ltd.) was administered via drinking water (0.3 mg/kg/day) during the age of 8–24 weeks for the *Fbn1*^C1041G/+^ mice with HFD (53). Transthoracic ultrasound imaging was performed using a Vevo 2100 system with a 30-MHz probe (FUJIFILM Visualsonics, Inc.). The mice were anesthetized with 2–3% isoflurane inhalation during ultrasound imaging assessment. A 2-dimensional long-axis view of the aorta was obtained to measure the aortic diameters at the level of the aortic root and AA. The acquisition and analysis of ultrasound imaging in this study were conducted by an independent operator blinded to the treatment/genotype of the animals. The blood pressure was noninvasively measured in conscious mice using the tail-cuff system (MK-2000ST NP-NIBP Monitor; Muromachi Kikai Co., Ltd.). Serum concentrations of total cholesterol, and blood sugar were measured in the laboratory of SRL, Inc.

### Western blot analysis.

Aortic tissues were harvested from the mice, and perivascular tissues were thoroughly removed from adventitia. Both tissues were used for western blot analysis. The aortas were divided into ascending thoracic (from the aortic root to immediately past the left subclavian artery) and descending thoracic aortas. Murine aortas and perivascular tissues were crushed by cryopress and dissolved in RIPA lysis buffer (50 mM Tris-HCl, pH 8.0, 150 mM NaCl, 1% NP-40, 1% SDS, 0.5% Na-deoxycholate, 10 mM Okadaic acid) containing a protease inhibitor cocktail (cOmplete ULTRA Tablets Mini EASYpack; Roche Diagnostics). Protein concentration was measured using a BCA Protein Assay Kit (Thermo Fisher Scientific). The lysates were fractionated with SDS–PAGE and transferred to polyvinylidene difluoride membrane (Merck Millipore). The blotted membranes were incubated with the following primary antibodies: rabbit monoclonal antiphosphorylated SMAD2 (Ser465/467) antibody (Cell Signaling Technology, #3108), rabbit monoclonal anti-SMAD2 antibody (Cell Signaling Technology, #5339), rabbit polyclonal antiphosphorylated ERK1/2 (Thr202/Tyr204) antibody (Cell Signaling Technology, #9101), rabbit polyclonal anti-ERK1/2 antibody (Cell Signaling Technology, #9102), rabbit polyclonal anti-IL-1β antibody (Cell Signaling Technology, #2022), mouse polyclonal anti-MCP-1 antibody (Cell Signaling Technology, #2029), mouse monoclonal anti-MMP-2 antibody (Abcam, #86607), rabbit recombinant multiclonal anti-MMP-9 antibody (Abcam, #283575), mouse monoclonal anti-β actin antibody (Sigma-Aldrich, #A2228). The membranes were then incubated with horseradish peroxidase-conjugated anti-mouse (Jackson Immuno Research Laboratories, Inc., #115-035-146) or anti-rabbit IgG antibody (Jackson Immuno Research Laboratories, Inc., #111-035-003). Immunoreactive signals were detected with ECL Prime Western Blotting Detection Reagent (GE Healthcare Biosciences) and visualized using the Lumino Graph I (ATTO Corp.). The images were converted to grayscale, and the integrated density per image area of interest was quantified using an NIH Image J software (NIH, Research Branch).

### Quantitative PCR (qPCR).

Aortic tissues were harvested from the mice, and perivascular tissues were thoroughly removed from adventitia. Perivascular tissues were used for reverse transcription PCR (RT-PCR). Total RNA from murine aortas and perivascular tissues was extracted using the RNeasy Mini Kit (Qiagen). Single-stranded cDNA was reverse transcribed using Superscript VILO cDNA synthesis kit (Invitrogen). qPCR was performed on QuantStudio 5 Real-Time PCR System (Thermo Fisher Scientific) using Thunderbird SYBR qPCR Mix (Toyobo). The specific primers are listed in Supplemental Table 1. Relative expression levels of target genes were calculated using the comparative CT method. Each sample was run in duplicate, and the results were systematically normalized using glyceraldehyde-3-phosphate dehydrogenase (*Gapdh*) or β-2 microglobulin (*B2m*) for experiments using mouse tissues.

### Histological and IHC analysis.

For histological analysis, the AA with perivascular tissues was excised, fixed immediately in 10% neutralized formalin (Sakura Finetek Japan Co., Ltd.), and embedded in paraffin. All paraffin-fixed specimens in each graph were processed in the same procedure. The anatomical location of the specimens that were analyzed to obtain the data ranged from the aortic valve up to 2,000 μm. Serial sections at 5 μm were stained with hematoxylin–eosin (H&E) for morphological analysis, elastica van Gieson (EVG) for detection of elastic fibers, Masson’s trichrome for detection of collagen fibers, and Alcian blue for staining proteoglycan. Degeneration of elastic fibers was evaluated by calculating the number of elastic fiber breaks per high power field (54). Breaks of elastic fiber and deposition of collagen were assessed at 4–5 different representative locations and averaged by 2 observers blinded to the genotype and treatment of each mouse. For IHC analysis, deparaffinized sections were rehydrated, and boiled to retrieve antigens (10 mM citrate buffer, pH 6.0 for staining of phosphorylated SMAD2 and ERK1/2; 0.1% trypsine solution for staining of F4/80). After blocking with 10% goat or rabbit serum in PBS and with Avidin/Biotin Blocking Kit (Vector Laboratories, Inc.), the sections were incubated with the following primary antibodies: rabbit monoclonal antiphosphorylated SMAD2 antibody (Ser465/467) (Cell Signaling Technology, #3108), rabbit polyclonal antiphosphorylated ERK1/2 antibody (Thr202/Tyr 204) (Cell Signaling Technology, #9101), and rat monoclonal anti-F4/80 antibody (Bio-Rad Laboratories, Inc., MCA497GA). Vectastain ABC kit (Vector Laboratories, Inc.) and DAB Peroxidase Substrate Kit (Vector Laboratories, Inc.) were used to detect the primary antibodies. The sections were counterstained with hematoxylin and mounted in Mount-Quick (Daido Sangyo Co., Ltd.). IHC staining for CD68 was performed with human aorta. Mouse monoclonal anti-human CD68 antibody (Dako, M0876) was used for staining whole macrophages with the same method as performed on mouse tissue. The ratio of F4/80-, CD68-positive cells to total PVAT area, and p-SMAD2-positive cells to total media area, determined from 3 random fields at 20× magnification, and the average percentage was used as the individual value by 2 observers blinded to genotype (26, 32, 55). Three images per aortic section were taken randomly and the pERK1/2 positive signal in media area was scored by 2 blinded observers. The staining was graded according to the following scale: 0, no staining; 1, minimal; 2, mild; 3, moderate; 4, maximal (56). To ensure that the staining of the tissue is a production of authentic recognition of the antigen, immunostaining was performed using negative and positive controls.

### Assessment of in situ MMP activity.

We measured gelatinase (MMP-2/gelatinase-A and MMP-9/gelatinase-B) activity by zymography, as described previously (57). Fluorescein-conjugated DQ Gelatin from Pig Skin (50 μg/mL, Thermo Fisher Scientific) in reaction buffer (0.5 M Tris-HCl, pH 7.6, 1.5 M NaCl, 50 mM CaCl_2_, 2 mM NaN_3_) was applied to freshly frozen sections (5 μm) and incubated at 37°C for 24 hours. The sections for negative control were incubated in the presence of 5-mM EDTA. The sections were stained and mounted in ProLong Gold Antifade Reagent with DAPI (Thermo Fisher Scientific). An all-in-one fluorescence microscope (BZ-9000; Keyence) was used to detect gelatinase (MMP-2 and MMP-9) activity via green fluorescence. The positive area of MMP activity to total media area, determined from 3 random fields at 20× magnification, and the average percentage was used as the individual value by 2 observers blinded to genotype (58).

### Human aortic tissue specimens.

This study was approved by the Research Ethics Committee, Graduate School of Medicine and Faculty of Medicine, The University of Tokyo [reference no. 2233-(10), G1538-(15)]. Ascending aortic aneurysm samples were collected from patients with MFS undergoing prophylactic aortic root surgery (*n* = 10; male, 7; age, 30.2 ± 10.3; the sinus of Valsalva: 42.1 ± 2.5 mm). All patients fulfilled MFS diagnostic criteria according to the revised Ghent nosology (59). Control aortic tissues were obtained from heart transplant recipients undergoing transplantation after obtaining written informed consent (*n* = 3; male, 2; age, 55.3 ± 2.6). Aortic tissue from heart transplant donors could not be used as a control tissue due to practical difficulties in obtaining consent from the donor’s family. To evaluate histological and immunohistochemical analysis, aortic tissues were either fixed in 10% formalin (Sakura Finetek Japan) and embedded in paraffin. All paraffin-fixed specimens in each graph were processed in the same manner.

### Study population.

MFS and LDS patients with indications for prophylactic aortic root and ascending aortic aneurysm surgery were selected as the disease group. All patients with MFS fulfilled diagnostic criteria according to the revised Ghent nosology (59). TGF-β-related genes were identified in all LDS patients with physical features similar to those in MFS. Random cases of thoracic contrast CT imaging with or without ascending aortic aneurysm, and cases in which ascending aortic aneurysm surgery was performed by cardiac surgery at our hospital and collaborating institutions were selected as the non-HCTDs group. Although patients in the non-HCTDs group have no physical characteristics suggestive of CTDs, genetic testing was not performed, which did not completely exclude nonsyndromic hereditary thoracic aortic aneurysm dissection. Cases of chronic aortic dissection, postoperative cases for aortic dissection, and cases that were difficult to analyze by machine learning were excluded. A multi-institutional study was conducted on patients without aortic aneurysms as comorbidities (mainly breast cancer patients and liver cancer patients). Patients who were difficult to analyze by machine learning were excluded.

### Image acquisition and data preparation.

All CT images were acquired from one of the following scanners with a resolution of 512 × 512 pixels, 5-mm slice thickness, and 120 kVp tube voltage: Aquilion Precision, Aquilion PRIME, Aquilion Prime SP, Aquilion ONE, or Aquilion (Canon Medical Systems); Discovery CT750 HD, Revolution CT, Revolution Frontier, LightSpeed, LightSpeed QX/i, or LightSpeed VCT (GE Healthcare); or Brilliance16 (Philips). The images were stored in the Digital Imaging and Communications in Medicine (DICOM) format. The Pydicom library was used to read and process DICOM information (60).

### AA-FAI.

AA-FAI analysis was performed using human CT images to evaluate vascular inflammation in patients with MFS. The region of the AA was annotated using Computer Vision Annotation Tool (CVAT), a labeling tool for computer vision algorithms. Ten patients’ CT scan slices were used to train a deep convolutional neural network (CNN) model based on Detectron2, an open-source modular object detection library. Our model was based on pretrained Mask R-CNN using ResNet conv4 backbone with a conv5 head (61). Mask R-CNN was trained on the COCO dataset (62), and the backbone model was set up based on ImageNet classification tasks (63). Transfer learning techniques were used to fine-tune the model. The optimizer used in this study was stochastic gradient descent (64) and the learning rate was set to 0.0001. Each training was conducted for 5,000 iterations with a batch size of 4. Only contrast-enhanced CT images were included in the analysis. The AA up to 5 cm distal to the aortic root was the target of the analysis. In a 10 mm region around the AA automatically recognized by machine learning, areas with CT values of –190 to 0 Hounsfield units (CT range of fat) were identified as perivascular fat. The specific setting of the CT range for fat may vary depending on the study models (34, 65), and in this study, we have defined the CT range for fat as –190 to 0 HU, taking into account the robust inflammation observed in the vessels of MFS. The CT values of each pixel in the perivascular fat were summed and divided by the number of pixels to calculate the mean CT value of the perivascular fat in each case.

### Definitions.

Hypertension was defined as a systolic blood pressure of > 140 mmHg, diastolic blood pressure of > 90 mmHg, or the use of medications for hypertension. Diabetes mellitus was defined as a hemoglobin A1c level of > 6.5% in National Glycohemoglobin Standardization Program units or the use of medications for diabetes mellitus. Dyslipidemia was defined as a low-density lipoprotein cholesterol level of > 140 mg/dL or the use of lipid-lowering medications. CKD was indicated by estimated glomerular filtration rate of < 60 mL/min/1.73 m^2^. Hyperuricemia was defined as a uric acid level of > 7.0 mg/dL or the use of uric acid-lowering medications. This study defined the degree of dilatation of ascending aortic diameter as follows, since there is no clear definition of ascending aortic dilatation. “Severe dilatation” is defined by the vessel diameter for which surgery is recommended (17), and “no dilatation” is defined by considering the definitions of the previous study (66). Body surface area was calculated using the Mosteller formula (SQRT ([Height(cm) *×* Weight(kg)]/3,600)). The ASI equals the aorta diameter (mm) divided by body surface area (m^2^) (44).

### Statistics.

Statistical analysis was performed using GraphPad Prism version 9.4.1 (GraphPad Software). All animal experiments and quantitative evaluation of human IHC staining are expressed as mean ± SEM and clinical data as mean ± SD. Kolmogorov-Smirnov test or D’Agostino-Pearson omnibus test were used for normality testing. Statistical significance was determined by either unpaired 2-tailed with Welch’s correction, 1-way ANOVA followed by Tukey’s multiple-comparison test, and 2-way ANOVA followed by Tukey’s multiple-comparison test. If the normality assumption was violated, nonparametric tests by Kruskal-Wallis test with Dunn’s multiple comparisons were conducted to identify differences between specific groups. Pearson’s chi-square test was used for comparison between 2 categorical variables. A ROC analysis was performed to evaluate the ability of AA-FAI parameters to discriminate between non-HCTDs and HCTDs or MFS. The optimal cut-off value was obtained using the Youden index from the ROC analysis. Values of *P* < 0.05 were considered statistically significant.

### Study approval.

All animal experiments were approved by the Animal Care Committee of The University of Tokyo (approval ID P-14-083 and P-20-040) and carried out in accordance with the animal experiment guidelines. The study followed the principles outlined in the Declaration of Helsinki. This study was approved by the ethics committee of the University of Tokyo (approval ID-2020019NI) and Kanto Central Hospital (approval ID-R4-35), and written informed consent was waived because this is a retrospective study using existing data. Instead, we used an opt-out approach to provide participants with an informed choice about participation, although no patient in the cohort for screening used the opt-out option. CT images were selected from July 2003 to October 2022, and the most recent of these images were selected for each patient.

### Data availability.

The data underlying this article will be shared upon reasonable request to the corresponding authors. We have provided Supporting Data Values associated with the main manuscript and supplemental material, including values for all data points shown in graphs and values behind any reported means.

## Author contributions

KU, HA, and IK conceived and designed the project. HS and H Yagi performed most of the experiments and analyzed data, with contributions from QL and AK, who provided experimental materials. MH, YA, TY, SO, BZ, NT, MA, H Yamauchi, KK, NI, and MO helped in the analysis and interpretation of data. HS, HY, KU, HA, and IK wrote the manuscript. All authors read and approved the manuscript. HS, H Yagi, and KU share the first-author position in the given sequence for their specific contributions based on workload and significance to the project.

## Supplementary Material

Supplemental data

Unedited blot and gel images

Supporting data values

## Figures and Tables

**Table 1:**
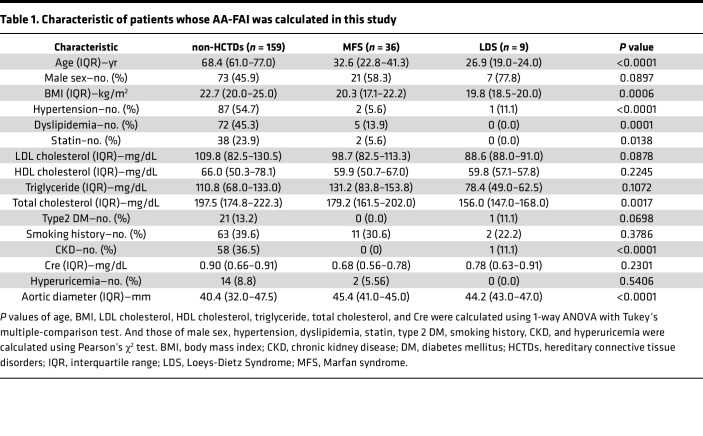
Characteristic of patients whose AA-FAI was calculated in this study

## Version 1 08/28/2025

In-Press Preview

## Version 2 10/08/2025

Electronic publication
