# Supplementary material for: Perivascular inflammation in the progression of aortic aneurysms in Marfan syndrome
Source: JCI Insight. 2025 Aug 28;10(19):e184329. doi: 10.1172/jci.insight.184329 (PMC12513498; doi:10.1172/jci.insight.184329)
Supplement: Supplemental data [file jciinsight-10-184329-s118.pdf]

Supplemental Material

Supplemental Figure 1. Systemic effects of HFD and pitavastatin on metabolic parameters.

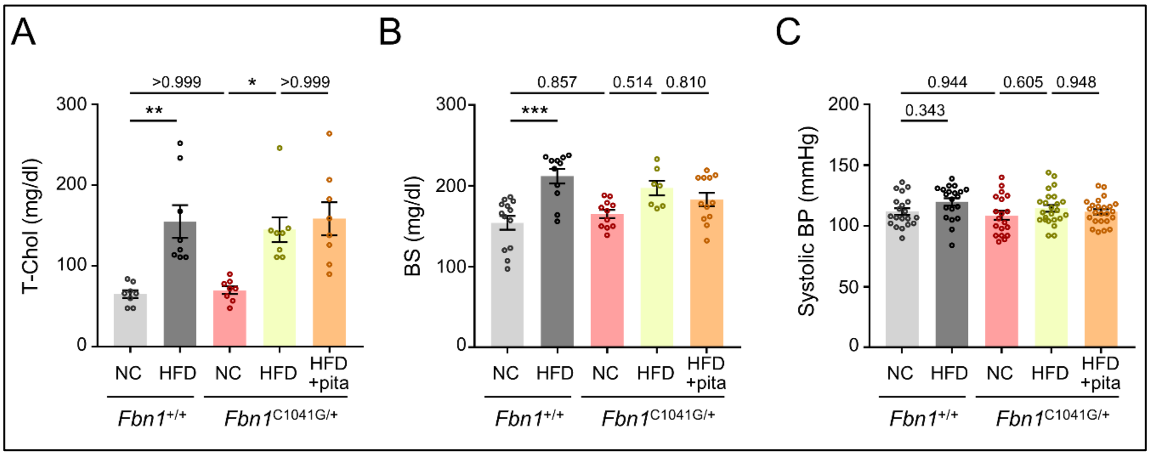

(**A-C**) Serum total-cholesterol (T-chol) (*n* = 8), blood sugar (BS) (*n* = 7 to 13) and systolic blood pressure (BP) (*n* = 19 to 24) in *Fbn1*$^{+/+}$ and *Fbn1*$^{C1041G/+}$ mice (24 weeks of age) receiving normal chow (NC) or high fat diet (HFD) or HFD with pitavastatin (pita). The data are presented as mean ± SEM. ***P*<0.01, ****P*<0.001, one-way ANOVA with Tukey's multiple comparisons test (middle and right panel) and Kruskal-Wallis test with Dunn’s multiple comparisons test (left pannel).

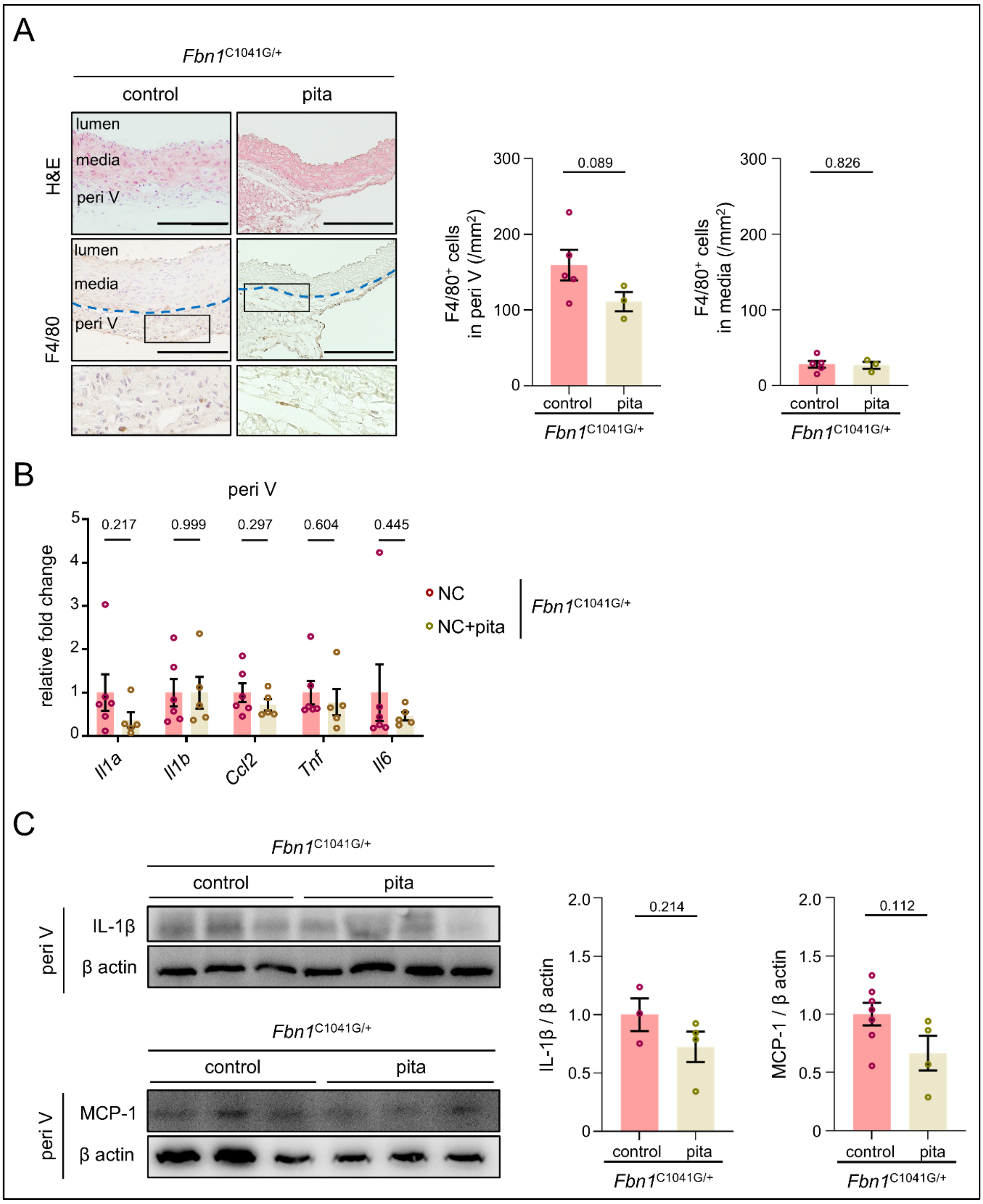

Supplemental Figure 2. Inflammation in PVAT on aortic dilatation in *Fbn1*$^{C1041G/+}$ mice.

(**A**) Immunohistochemical staining for F4/80 in ascending aorta of *Fbn1*$^{C1041G/+}$ mice (24 weeks of age) receiving normal chow (NC) with or without pitavastatin (pita) (*n* = 3 to 5). Scale bars,

200 μm. Blue dashed lines indicate the external elastic lamina. The data are presented as mean ± SEM. Unpaired 2-tailed t test with Welch's correction. (**B**) The mRNA expressions of marker genes of inflammatory cytokines in the peri vascular tissues (peri V) of *Fbn1*$^{+/+}$ and *Fbn1*$^{C1041G/+}$ mice (24 weeks of age) receiving NC with or without pita (*n* = 5 to 6). The data are presented as fold induction over control. The results were systematically normalized using β-2 microglobulin (*B2m*). The data are presented as mean ± SEM. Unpaired 2-tailed t test with Welch's correction. (**C**) Immunoblot analysis of IL-1β, MCP-1, and β actin in peri V of *Fbn1*$^{+/+}$ and *Fbn1*$^{C1041G/+}$ mice (24 weeks of age) receiving NC with or without pita. The quantifications of IL-1β/β actin (*n* = 3 to 4) and MCP-1/β actin (*n* = 4 to 7) are shown as bar graphs. The data are presented as mean ± SEM. Unpaired 2-tailed t test with Welch's correction.

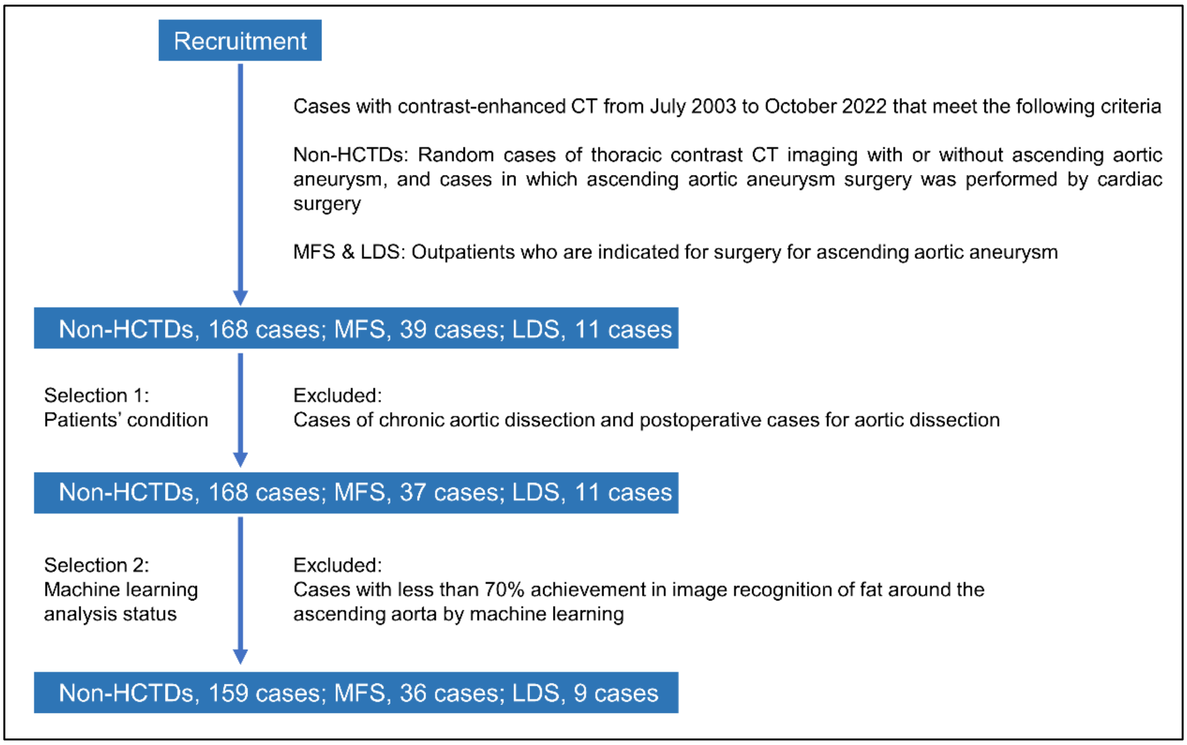

Supplemental Figure 3. Study flow

The source population included 218 patients, two MFS patients were excluded due to patients' condition, and nine non-HCTDs patients and one MFS patient and two LDS patients were excluded due to machine learning analysis status. The remaining 204 patients (non-HCTDs, n = 159; MFS, n = 36; LDS, n = 9) were eligible. CT, computed tomography; HCTDs, hereditary connective tissue disorders; LDS, Loeys–Dietz Syndrome; MFS, Marfan syndrome.

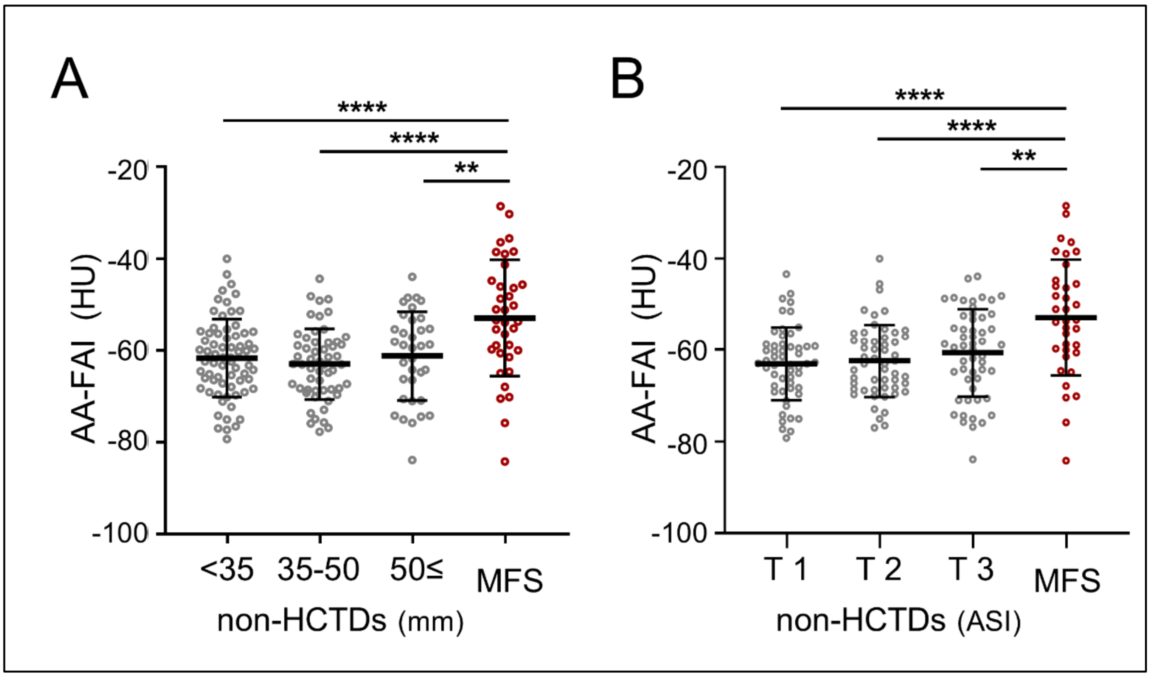

Supplemental Figure 4. AA-FAI and aortic dilatation

(A) The fat attenuation index of perivascular adipose tissue surrounding the ascending aorta (AA-FAI) was calculated in non-HCTDs group (no aortic dilatation <35mm, n = 69, mild aortic dilatation 35-49mm, n = 55, severe aortic dilatation 50≤mm, n = 35) and MFS group (n = 36). The data are presented as mean ± SD. **P<0.01, ****P<0.0001, one-way ANOVA with Tukey's multiple comparisons test. (B) The non-HCTDs group was further divided into tertiles based on aortic size index (ASI) (n = 53 per tertile): lower tertile (T1, mean ASI 19.11), middle tertile (T2, mean ASI 23.22), and upper tertile (T3, mean ASI 33.98). The AA-FAI in MFS patients (n = 36, mean ASI 25.41) was significantly higher than in any of the non-HCTD tertiles. Data are presented as mean ± SD. **P<0.01, ****P<0.0001, one-way ANOVA with Tukey’s multiple comparisons test. HCTDs, hereditary connective tissue disorders; MFS, Marfan syndrome.

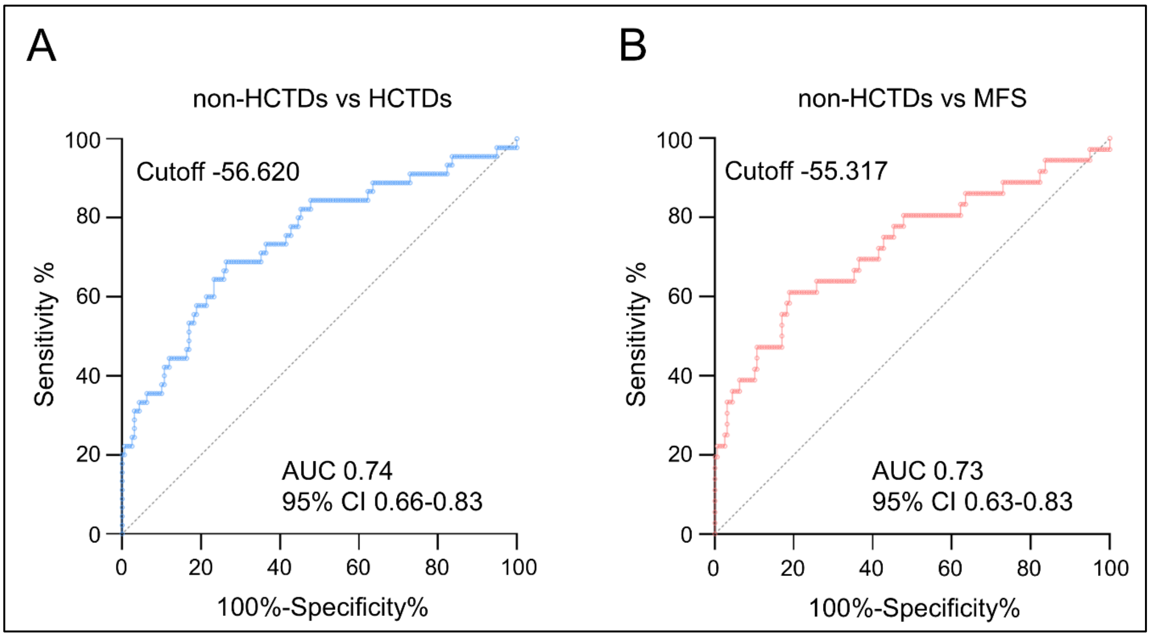

Supplemental Figure 5. Receiver operating characteristic (ROC) analysis on AA-FAI of prediction for HCTDs or MFS.

(A) Non-HCTDs (*n* = 159) vs HCTDs (*n* = 45). (B) non-HCTDs (*n* = 159) vs MFS (*n* = 36). AUC; area under the curve, HCTDs; hereditary connective tissue disorders, MFS; Marfan syndrome.

Supplemental Table 1. List of sequences of Real-time quantitative RT-PCR primers.

Genes	Primer forward	Primer reverse
Adgre1	CTTTGGCTATGGGCTTCCAGTCC	GCAAGGAGGACAGAGTTTATCGTG
B2m	CATGGCTCGCTCGGTGAC	CAGTTCAGTATGTTCGGCTTCC
Ccl2	CCACTCACCTGCTGCTACTCAT	TGGTGATCCTCTTGTAGCTCTCC
Cd163	TCCACACGTCCAGAACAGTC	CCTTGGAAACAGAGACAGGC
Cd68	ACTTCGGGCCATGTTTCTCT	GCTGGTAGGTTGATTGTCGT
Gapdh	CACTGAAGGGCATCTTGG	CATTGTCATACCAGGAAATGAG
Il1a	TTGGTTAAATGACCTGCAACA	GAGCGCTCACGAACAGTTG
Il1b	AGTTGACGGACCCCAAAAG	AGCTGGATGCTCTCATCAGG
Il6	CTGCAAGAGACTTCCATCCAG	AGTGGTATAGACAGGTCTGTTGG
Mrc1	CAGGTGTGGGCTCAGGTAGT	TGTGGTGAGCTGAAAGGTGA
Serpine1	CCTCCTCATCCTGCCTAAGTT	GGCCAGGGTTGCACTAAAC
Tnf	TCTTCTCATTCCTGCTTGTGG	GAGGCCATTTGGGAACTTCT

Supplemental Table 2. Characteristic of patients with non-HCTD

Characteristic	No dilatation (<35mm) (n=69)	Mild dilatation (35-49mm) (n=55)	Severe dilatation (50mm≤) (n=35)
Age (IQR) -yr	69.6 (61.0-77.0)	71.2 (66.5-78.5)	61.7 (55.0-71.5)
Male sex-no. (%)	14 (20.3)	33 (60.0)	26 (74.3)
BMI (IQR)-kg/m^2	22.1 (19.8-24.3)	23.9 (20.3-26.3)	22.0 (20.0-23.9)
Hypertension-no. (%)	32 (46.4)	32 (58.2)	23 (65.7)
Dyslipidemia-no. (%)	37 (53.6)	26 (47.3)	9 (25.7)
Statin-no. (%)	17 (24.6)	15 (27.3)	6 (17.1)
LDL cholesterol (IQR)-mg/dL	114.3 (86.0-138.8)	105.5 (72.0-125.5)	107.8 (93.5-122.8)
HDL cholesterol (IQR)-mg/dL	74.9 (60.2-86.7)	58.7 (48.4-64.9)	59.6 (47.5-66.4)
Triglyceride (IQR)-mg/dL	98.4 (64.0-119.0)	122.7 (65.3-148.0)	114.8 (80.8-133.5)
Total cholesterol (IQR)-mg/dL	209.0 (181.0-238.0)	187.8 (169.0-205.0)	190.8 (169.0-214.8)
Type2 DM-no. (%)	12 (17.4)	7 (12.7)	2 (5.7)
Smoking history-no. (%)	20 (29.0)	25 (45.5)	16 (45.7)
CKD-no. (%)	25 (36.2)	23 (41.8)	10 (28.6)
Cre (IQR)-mg/dL	0.75 (0.62-0.84)	0.93 (0.71-0.98)	1.16 (0.73-0.95)
Hyperuricemia-no. (%)	6 (8.7)	5 (9.1)	3 (8.6)
Aortic diameter (mm)	30.9 (30.0-33.0)	39.9 (36.5-42.0)	59.9 (55.5-65.0)

BMI, body mass index; CKD, chronic kidney disease; DM, diabetes mellitus; IQR, Interquartile range
