# Supplementary figures and images for: Perivascular inflammation in the progression of aortic aneurysms in Marfan syndrome
Source: JCI Insight. 2025 Aug 28;10(19):e184329. doi: 10.1172/jci.insight.184329 (PMC12513498; doi:10.1172/jci.insight.184329)

Figure 1

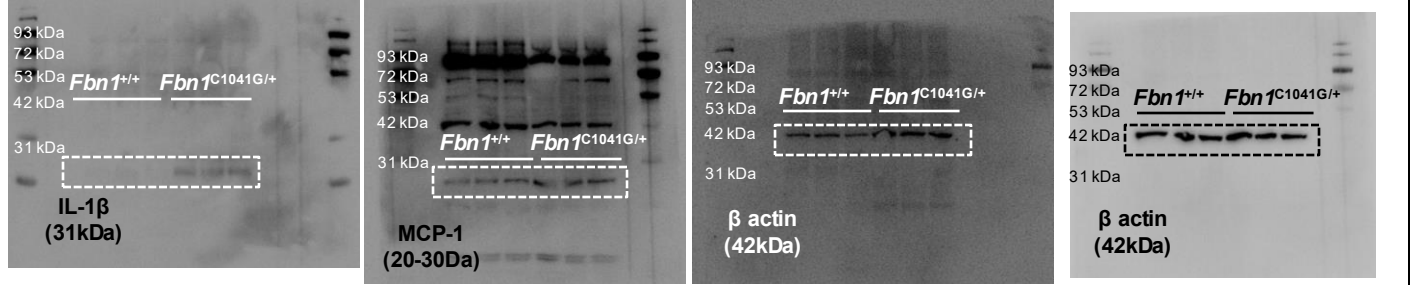

Figure 2

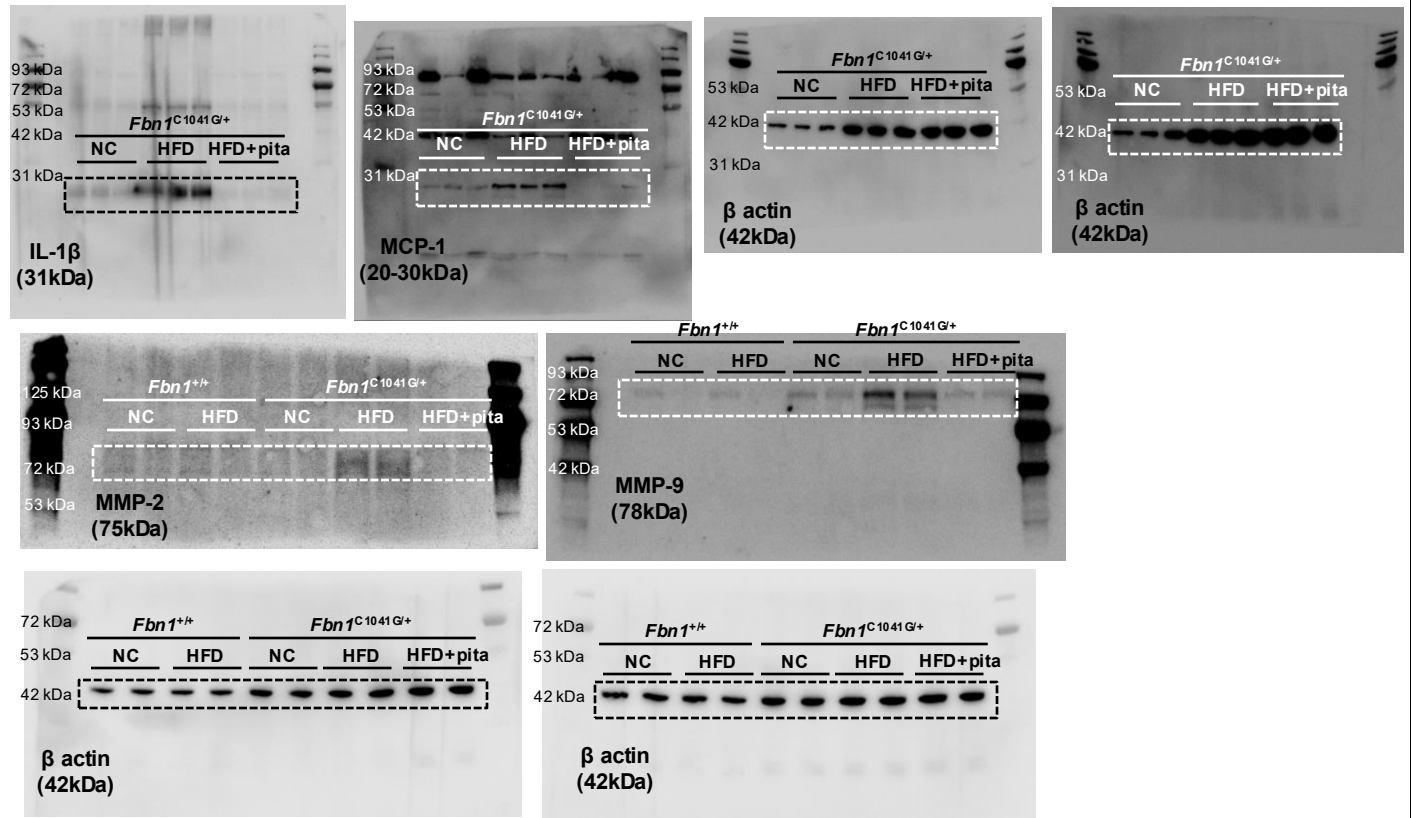

Figure 4

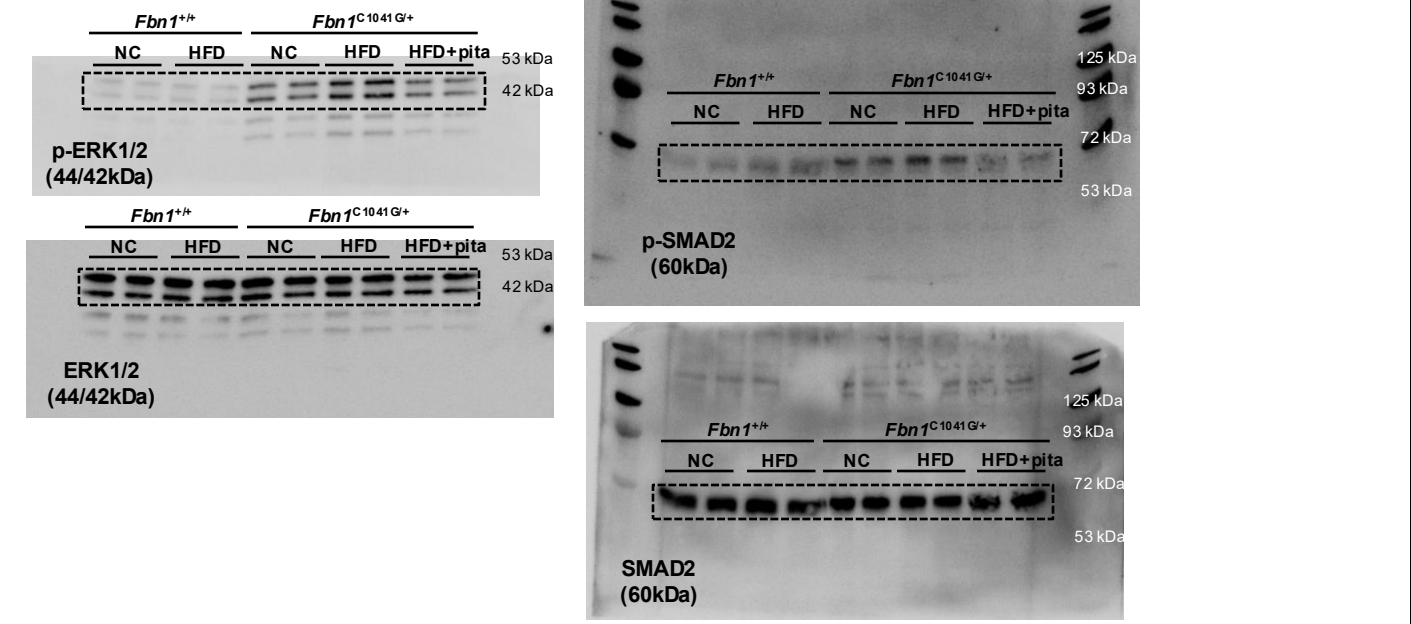

Supplemental Figure 2

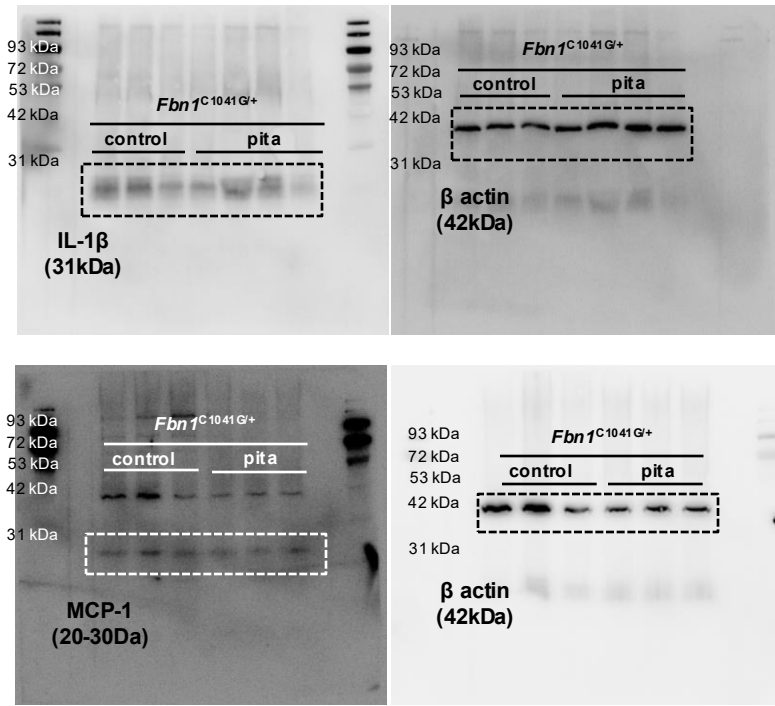

Supplement: Unedited blot and gel images [file jciinsight-10-184329-s119.pdf]
